# Gender and risk‐taking behaviors influence the clinical presentation of oral squamous cell carcinoma

**DOI:** 10.1002/cre2.523

**Published:** 2022-01-05

**Authors:** Susanne Wolfer, Annika Kunzler, Tatjana Foos, Cornelia Ernst, Andreas Leha, Stefan Schultze‐Mosgau

**Affiliations:** ^1^ Department of Oral and Maxillofacial & Plastic Surgery Jena University Hospital Jena Germany; ^2^ Department of Medical Statistics University Medical Center Goettingen Goettingen Germany

**Keywords:** alcohol, gender, oral squamous cell carcinoma, smoking

## Abstract

**Objective:**

The common risk factors for oral squamous cell carcinoma (OSCC) are smoking and alcohol abuse. A small percentage of patients, mostly women, are demonstrating oral cancer without the common risk behavior. This study investigates how gender and different patterns of lifestyle factors influence the clinical presentation of OSCC.

**Patients and Methods:**

From this retrospective study, demographical and tumor‐specific data and lifestyle factors were analyzed. Statistical analyses were performed using the *χ* ^2^ test or Fisher's exact test for categorical analysis and the *t* test, ANOVA test, or Kruskal–Wallis test for continuous variables. The influence of the respective lifestyle factors together with their interactions with the gender on tumor characteristics has been tested using logistic and ordinal cumulative link regression models.

**Results:**

Among a total of 308 patients, men represented the majority of smokers (87.2%) and the female cohort were largely non‐smokers and non‐drinkers (64.9%). For age, tumor site and N‐stage it looks like that differences of men and women are driven by the different risk behavior. But if the lifestyle factors are taken into account, we observe contrary effects between men and women for T‐, N‐, and UICC‐stage. For different cancer locations we saw opposite effects with gender and risk profile. These effects are not dose‐dependent explainable for gender.

**Conclusion:**

Some but not all differences in the development of OSCC for men and women are explainable by the respective difference in lifestyle behavior. Some further investigations are necessary to find explanations for the obvious differences between men and women in developing OSCC.

## 1 INTRODUCTION

Cancers of the oral cavity and the lips are common malignant tumors of the head and neck. These cancers accounted for approximately 2% of all cancers and cancer deaths worldwide, with an increase from 300,000 to 350,000 new cases and from 145,000 to 177,000 deaths in 2012 and 2018. (Bray et al., 2018; Ferlay et al., 2015; Torre et al., 2015) Cancers of the oral cavity had the highest frequency, with approximately 200,000 cases (Shield et al., 2017). In Germany, approximately 13,000 people per year develop cancers of the oral cavity, and more than 95% of them are squamous cell carcinomas (Wolff et al., 2012). However, oral cavity cancer can be divided into multiple tumor locations, such as floor of the mouth (FOM), tongue, alveolar rim, palate, and buccal mucosa. Oral squamous cell carcinomas (OSCC) are found more frequently in men than in women, and it is widely recognized that there is a strong association between oral cancer and smoking and alcohol consumption (Bray et al., 2018; Ferlay et al., 2015; Poeschl & Seitz, 2004; Shaw & Beasley, 2016; Shield et al., 2017; Wolff et al., 2012). In many studies, factors such as gender and risk behavior in relation to the development of OSCC were considered, but considered separately (Pires et al., 2013; Shaw & Beasley, 2016). It is well known that men make up the greater proportion of patients and also display more risk behavior. And many women show no risk behavior at diagnosis. It was also shown, that women even exhibited larger increasing changes in incidence compared with men (Chaturvedi et al., 2013; Du et al., 2020). Investigating the differences of men and women should include a more detailed analysis of the well‐known factors. Thus, the interactions of the known risk factors with the sexes should be examined in order to get to the bottom of the obvious difference in development of OSCC between men and women and also to determine equality.

The aim of this study is therefore to analyze the demographic and clinical data, with a focus on gender, and lifestyle in general and in specific to the interaction effect of risk behavior and gender to get information how it influence the clinical presentation of cancers, especially in the oral cavity.

## 2 PATIENTS AND METHODS

The investigators designed and implemented a retrospective clinic‐statistical study. The study population was composed of all patients presenting for surgical treatment for OSCC at a single department over a ten‐year period between May 2005 and April 2015. To be included in this study sample, patients had to meet the following inclusion criteria: (1) first diagnosed and histologically confirmed OSCC between May 2005 and April 2015; (2) tumor location on the FOM, oral tongue, alveolar rim, palate, buccal mucosa, or advanced tumors that affect more than one region. Exclusion criteria were (1) oro‐, naso‐, and hypopharyngeal cancers and lip cancers; (2) treatment not within the period under review, and (3) insufficient documentation. The data extracted from medical records were analyzed for gender, age, tumor size, tumor location, tumor differentiation, N‐stage, Union for International Cancer Control (UICC) stage, tobacco use, and alcohol consumption.

In this study, the investigators maintained a particular focus on the different risk behaviors. Lifestyle behavior with tobacco and alcohol consumption was collected from patients' self‐reported information on medical records and categorized into four groups: non‐smokers/non‐drinkers (NSND); smokers only (smokers and non‐drinkers [SND]), drinkers only (non‐smokers and drinkers [NSD]), and smokers/drinkers (SD) as consumers of both. The intensity of smoking was measured with pack years (cigarettes packs per day × years smoked); one pack year is a regularly smoking of 20 cigarettes (=1 pack) daily for 1 year or regular smoking 10 cigarettes per day for 2 years or 40 cigarettes per day for 6 months. No drinking was defined as occasionally drinking alcohol within recommended limits or drinking no alcohol at all. The reported age was the patient's age at diagnosis. The stage classification was performed according to the seventh edition of the UICC (Union Internationale Contre le Cancer). All included patients were white Europeans.

All variables have been summarized by absolute and relative frequencies or by mean ± standard deviation and median (minimum; maximum) as appropriate. Descriptive values have been generated for the full cohort as well as separately by gender as well as separately by risk behavior group. Variables have been compared univariately between genders using paired *t* test, *χ* ^2^ test, Mann–Whitney *U* test, or Fisher's exact test as appropriate and compared also univariately between risk behavior groups using ANOVA, Kruskal–Wallis test, *χ* ^2^ test, or Fisher's exact test as appropriate.

The influence of the risk factors together with their interactions with gender on tumor location, T‐stage, N‐stage, UICC‐stage, and differentiation have been tested using logistic and ordinal cumulative link models (Agresti, 2013). Resulting estimates (on the log scale) are reported with their 95% confidence intervals and associated *p* value. The modeled marginal effects have been predicted and visualized.

The significance level was set to *α *= 5% for all statistical tests. All analyses were performed with the statistic software R (Version 3.6.2) (R Core Team, 2018) using the R‐packages ordinal (Version 2019.12.10) (Christensen, 2019) for the cumulative link models and ggeffects (Version 0.14.1) (Lüdecke, 2019) for the visualization of the marginal effects. The study was approved with the reference number 4434‐05/15 in May 2015 by the local Ethics Committee.

## 3 RESULTS

### 3.1 Demographic analysis

A total of 308 patients with histologically confirmed OSCC, who met the inclusion criteria, were seen within the period under review in this study. The male‐to‐female ratio was 3:1, with 231 men and 77 women. The patients' ages ranged from 35 to 93 years, with a median of 58 years (mean 61 ± 12 years) in general. The male mean age was 59 ± 11 years, with a range from 35 years to 89 years. The female mean age was 65 ± 13 years, nearly 6 years higher than the male mean age (*p* < .01). The youngest woman was 38 years old and the oldest in this study was 93 years old. All demographic and clinical data regarding gender are shown in Table 1.

**Table 1:** Analysis of categorical and continuous variables regarding gender in patients with oral squamous cell carcinoma

Parameter	Total, *n*	Male, *n*	Female, *n*	*p* Value	Test
	308	231	77		
Age (years)				<.01	Welch two sample *t* test
Mean ± standard deviation	61 ± 12	59 ± 11	65 ± 13		
Median (min; max)	58 (35; 93)	56 (35; 89)	65 (38; 93)		
Location				.02	Pearson's *χ* ^2^ test
Palate	18 (5.8%)	12 (5.2%)	6 (7.8%)		
FOM	142 (46.11%)	118 (51.1%)	24 (31.2%)		
Buccal mucosa	8 (2.6%)	4 (1.7%)	4 (5.2%)		
Alveolar rim	44 (14.3%)	28 (12.1%)	16 (20.8%)		
Tongue	72 (23.4%)	49 (21.2%)	23 (29.9%)		
>One region	24 (7.8%)	20 (8.7%)	4 (5.2%)		
T‐status				.14	Wilcoxon's rank sum test with continuity correction
T1	107 (35.2%)	75 (32.6%)	32 (43.2%)		
T2	96 (31.6%)	74 (32.2%)	22 (29.7%)		
T3	33 (10.9%)	29 (12.6%)	4 (5.4%)		
T4	68 (22.7%)	52 (22.6%)	16 (21.6%)		
Missing	4	1	3		
N‐status				.01	Fisher's exact test for count data
N0	169 (56.0%)	120 (52.9%)	49 (65.3%)		
N1	48 (15.9%)	33 (14.5%)	15 (20.0%)		
N ≥ 2	85 (28.1%)	74 (32.6%)	11 (14.7%)		
Missing	6	4	2		
UICC‐status				.12	Wilcoxon's rank sum test with continuity correction
I	81 (27.0%)	56 (24.8%)	25 (33.8%)		
II	51 (17.0%)	40 (17.7%)	11 (14.9%)		
III	44 (14.7%)	31 (13.7%)	13 (17.6%)		
IV	124 (41.3%)	99 (43.8%)	25 (33.8%)		
Missing	8	5	3		
Differentiation				.44	Wilcoxon's rank sum test with continuity correction
Poor differentiated	75 (25.1%)	59 (26.1%)	16 (21.9%)		
Moderate differentiated	195 (65.2%)	146 (64.6%)	49 (67.1%)		
Well differentiated	29 (9.7%)	21 (9.3%)	8 (11.0%)		
Missing	9	5	4		
Smoking				<.01	Fisher's exact test for count data
No	105 (34.1%)	54 (23.4%)	51 (66.2%)		
Yes	203 (65.9%)	177 (76.6%)	26 (33.8%)		
Alcohol drinking				<.01	Fisher's exact test for count data
No	174 (56.5%)	106 (45.9%)	68 (88.3%)		
Yes	134 (43.5%)	125 (54.1%)	9 (11.7%)		
Risk behavior				<.01	Pearson's *χ* ^2^ test
NSND	95 (30.8%)	45 (19.5%)	50 (64.9%)		
SND	79 (25.6%)	61 (26.4%)	18 (23.4%)		
NSD	10 (3.2%)	9 (3.9%)	1 (1.3%)		
SD	124 (40.3%)	116 (50.2%)	8 (10.4%)		
Risk factors				<.01	Wilcoxon's rank sum test with continuity correction
Single	89 (28.9%)	70 (30.3%)	19 (24.7%)		
Double	124 (40.3%)	116 (50.2%)	8 (10.4%)		
Non	95 (30.8%)	45 (19.5%)	50 (64.9%)		
Pack years				1.00	Wilcoxon's rank sum test with continuity correction
Mean ± standard deviation	34 ± 14	34 ± 14	33 ± 12		
Median (min; max)	30 (5; 86)	30 (5;86)	30 (15; 50)		
Missing	56	47	9		

Abbreviations: CI, confidence interval; FOM, floor of the mouth; NSD, non‐smoker and drinker; NSND, non‐smokers and non‐drinkers; SD, smoker and drinker; SND, smokers and non‐drinker.

### 3.2 Tumor site

The majority of the tumors were located in the FOM (*n* = 142; 46.1%) and the oral tongue (*n* = 72; 23.4%). The remaining tumors were located in the alveolar rim (*n* = 44; 14.3%), palate (*n* = 18; 5.8%), and buccal mucosa (*n* = 8; 2.6%). In 7.8% (*n* = 24), tumors affected more than one region. In cancer location we saw differences for gender aspects (*p* = .02). In comparison to men, we saw that women were affected more by tongue cancers (29.9% vs. 21.2%) and alveolar rim cancers (20.8% vs. 12.1%). However, in both men and women, FOM was the main location, with 51.1% and 31.2%, respectively.

### 3.3 T‐ and N‐stage

Mostly T1 (35.2%) and T2 (31.6%) tumors were seen. More than half of the patients had N0 necks (56.0%). T‐stage showed no differences in gender whereas in N‐stage men had more N ≥ 2 than women who had mostly N0 stage (*p* = .01) (Figures S1 and S2).

### 3.4 UICC and tumor differentiation

Most OSCC were presented at diagnosis in UICC status IV with 41.3%. A high proportion of males presented UICC status IV with 43.8%, whereas women showed cancers with UICC status I with 33.8%. There were no statistical differences in gender regarding UICC distribution and tumor differentiation (Figure S3).

### 3.5 Tobacco and alcohol consumption

Among all patients, 213 patients (69.2%) had a tobacco‐ and/or alcohol‐positive history. We saw 177 men (76.6%) but only 26 women (33.8%) who were smokers (*p* < .01). However, within the group of smokers, there was no difference in gender in terms of the intensity measured by pack years (*p* = 1.00). Men reported a tobacco consumption of approximately 34 ± 14 pack years and women of 33 ± 12 pack years at diagnosis. So, men and women smoke equally intensely.

In general, 134 patients (43.5%) had a positive alcohol history. Like for tobacco use, there were significantly more men (*n* = 125; 54.1%) who consumed alcohol regularly than women (*n* = 9; 11.7%, *p* < .01).

Among all 308 patients, 95 patients were NSND (30.8%), 79 patients were SND (25.6%), 10 patients were NSD (3.2%) and 124 patients were SD (40.3%). The distribution according to gender, age, tumor location, T‐stage, N‐stage, UICC‐stage, and tumor differentiation for each group is shown in Table 2.

**Table 2:** Analysis of categorical and continuous variables regarding risk behavior in patients with oral squamous cell carcinoma

Parameter	Total, *n*	NSND, *n*	SND and NSD, *n*	SD, *n*	*p* Value	Test
	308	95 (30.8%)	89 (28.9%)	124 (40.3%)		
Gender					<.01	Fisher's exact test for count data
Male	231 (75.0%)	45 (47.4%)	70 (78.7%)	116 (93.5%)		
Female	77 (25.0%)	50 (52.6%)	19 (21.3%)	8 (6.5%)		
Age (years)					<.01	Analysis of variance
Mean ± standard deviation	61 ± 12	67 ± 13	60 ± 11	56 ± 9		
Median (min; max)	58 (35; 93)	68 (35; 93)	57 (37; 84)	54 (38; 78)		
Location					<.01	Pearson's *χ* ^2^ test
Palate	18 (5.8%)	4 (4.2%)	9 (10.1%)	5 (4.9%)		
FOM	142 (46.1%)	27 (28.4%)	41 (46.1%)	74 (59.7%)		
Buccal mucosa	8 (2.6%)	7 (7.4%)	1 (1.1%)	0 (0.0%)		
Alveolar rim	44 (14.3%)	20 (21.1%)	12 (13.5%)	12 (9.7%)		
Tongue	72 (23.4%)	32 (33.7%)	18 (20.2%)	22 (17.7%)		
>One region	24 (7.8%)	5 (5.3%)	8 (9.0%)	11 (8.9%)		
T‐stage					.22	Kruskal–Wallis rank sum test
T1	107 (35.2%)	39 (41.5%)	33 (37.9%)	35 (28.5%)		
T2	96 (31.6%)	27 (28.7%)	25 (28.7%)	44 (35.8%)		
T3	33 (10.9%)	10 (10.6%)	9 (10.3%)	14 (11.4%)		
T4	68 (22.6%)	18 (19.4%)	20 (23.0%)	30 (24.4%)		
Missing	4	1	2	1		
N‐stage					<.01	Fisher's exact test for count data
N0	169 (56.0%)	60 (65.2%)	50 (56.2%)	59 (48.8%)		
N1	48 (15.9%)	15 (16.3%)	20 (22.5%)	13 (10.7%)		
N ≥ 2	85 (28.1%)	17 (18.5%)	19 (21.3%)	49 (40.5%)		
Missing	6	3	0	3		
UICC stage					.08	Kruskal–Wallis rank sum test
I	81 (27.0%)	30 (32.6%)	27 (31.0%)	24 (19.8%)		
II	51 (17.0%)	16 (17.4%)	10 (11.5%)	25 (20.7%)		
III	44 (14.7%)	14 (15.2%)	17 (19.5%)	13 (10.4%)		
IV	124 (41.3%)	32 (34.8%)	33 (37.9%)	59 (48.8%)		
Missing	8	3	2	3		
Differentiation					.12	Kruskal–Wallis rank sum test
Poorly differentiated	75 (25.1%)	19 (20.4%)	23 (27.1%)	33 (27.3%)		
Moderately differentiated	195 (65.2%)	60 (64.5%)	53 (62.4%)	82 (67.8%)		
Well differentiated	29 (9.7%)	14 (15.1%)	9 (10.6%)	6 (5.0%)		
Missing	9	2	4	3		

Abbreviations: CI, confidence interval; FOM, floor of the mouth; NSD, non‐smoker and drinker; NSND, non‐smokers and non‐drinkers; SD, smoker and drinker; SND, smokers and non‐drinker.

**Figure 1:**
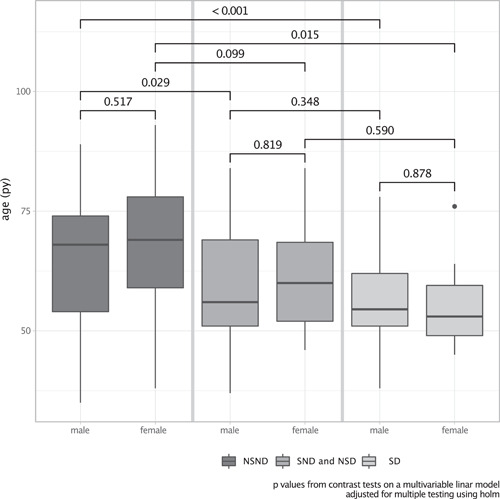
Different risk‐taking behavior according to the patients age (in years) divided for males and females: With increasing risk behavior age at diagnosis decreases, but with no differences in gender within the risk groups. NSD, non‐smoker and drinker; NSND, non‐smokers and non‐drinkers; SD, smoker and drinker; SND, smokers and non‐drinker

Females were more numerous in the NSND group (64.9%) whereas the majority of male patients present at least one risk factor (80.5%). We saw that SD patients had a mean age of 56 ± 9 years whereas the NSND patients had a mean age of 67 ± 13 years, respectively (*p* < .01). The groups with just one risk factor show a mean age of 60 ± 11 years. Therefore, with increasing risk behavior, the age decreases for OSCC patients. In gender‐separated analysis, we saw the same effect. Within the single risk groups, there were no differences regarding age between male and female patients (Figure 1).

Patients of different risk factor groups show significantly different tumor locations (*p* < .01). The main tumor location for patients with no risk factors (NSND) was the tongue with 33.7%. For patients with one (SND and NSD) or even two (SD) risk factors the main location was the FOM with 46.1% and 59.7%, respectively. For smoking and non‐smoking we see the same with no differences in gender (Figure S4).

Regarding N‐stage, patients with two risk factors developed more *N* ≥ 2 than did patients with no or one risk factor (*p* = .01). There was a tendency for more T1 in the NSND group but with no statistical differences in T‐stage between the risk groups. SD patients presented more UICC IV stages in comparison to NSND and SND and NSD patients but without reaching clear statistical significance (*p* = .08).

We fitted logistic regression models to predict each of the different main locations, FOM and tongue, with gender and the risk factors. For FOM in general the effect of the female gender is negative and the effect of smoking and alcohol is positive. Especially for women who smoke, the interaction effect to get a carcinoma at FOM is positive. The opposite was seen for tongue cancers. Here the effect for women to get tongue cancer is positive but in general for smoking and alcohol negative. The interaction effect for smoking women for tongue cancer is negative. None of these effects was significant (Table S1). There is also an opposite effect evident when comparing non‐smokers with smokers for FOM and tongue cancers (Table 3).

**Table 3:** Analysis of tumor site compared between FOM/tongue versus smoking/non‐smoking

Tumor sites	Group	Effect	*β*	95% CI	*p* Value
	m—non‐smoker	Reference			
FOM	m—smoker	+	.85	0.22; 1.48	.008
	f—non‐smoker	−	−.68	−1.55; 0.19	.126
	f—smoker	+	.61	−0.34; 1.56	.208
	m—non‐smoker	Reference			
Tongue	m—smoker	**−**	−.48	−1.18; 0.22	.180
	f—non‐smoker	+	.35	−0.48; 1.18	.408
	f—smoker	**−**	−.48	−1.62; 0.66	.411

*Note*: We fitted a logistic model to predict the tumor sites, FOM and tongue, with different risk behaviors. Male non‐smokers served as the reference group. Within this model —FOM: The effect of (m—smoker) is positive and can be considered as small and significant. The effect of (f—non‐smoker] is negative. The effect of (f—smoker) is positive. Tongue: The effect of (m—smoker) is negative. The effect of (f—non‐smoker) is positive. The effect of (f—smoker) is negative. See the adverse effects between the locations FOM and tongue. The model's intercept is at –0.61 for FOM and at –0.96 for the tongue.

Abbreviations: CI, confidence interval; f, female; FOM, floor of the mouth; m, male.

**Figure 2:**
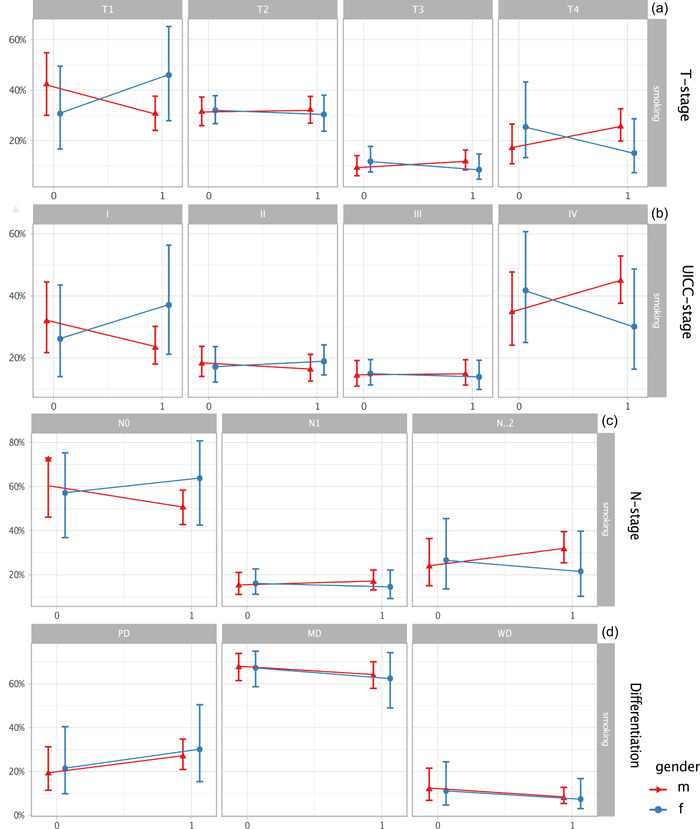
Smoking according to T‐stage, N‐stage, UICC‐stage, and differentiation divided as males and females: (a–c) There are opposite interaction effects visible for men and women for T1‐ and T4‐stage, for UICC‐stage I and IV and for N0‐ and N2‐stage (all with no statistical significance). In differentiation (d) there is no gender effect (ordinal cumulative [logit] link [proportional odds] model). f, female; m. male; MD, moderately differentiated; PD, poorly differentiated; WD, well differentiated; 0, no smoking; 1, smoking. For model coefficients and statistics see Table S2

The influence of the risk factors together with their interactions with the gender on T‐stage, N‐stage, UICC‐stage, and differentiation were investigated. In particular, for the risk factor of smoking, we see an adverse interaction effect for the T1 and T4 stage, for N0 and *N* ≥ 2 stage, and for UICC I and IV stage for men and women but without reaching statistical significance. Regarding differentiation, there were no differences of effects (Figure 2).

**Figure 3:**
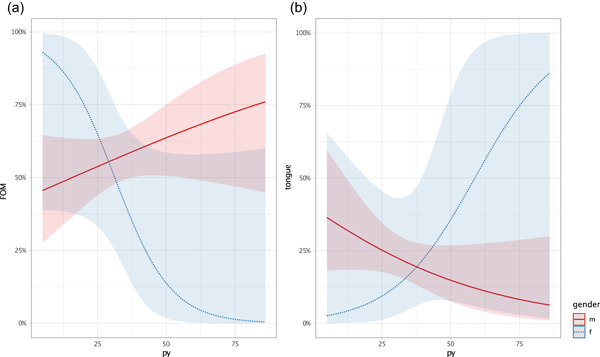
Pack years (py) broken down according to the tumor main locations, FOM and tongue, divided in gender. There is an opposite interaction effect in gender within FOM (a) and tongue (b) and an opposite effect between the locations, FOM and tongue, is visible (without statistical significance) (logistic regressions model). f, female; FOM, floor of the mouth; m, male (1 py = regular smoking 20 cigarettes (1 pack)/day for 1 year. With increasing py, there are more FOM cancers but less tongue cancer in smoking men. For women who smoke you can see the opposite effect. For statistics see Table S3

We saw some different effects in gender and lifestyle factors, but we further wanted to investigate the influence of the number of pack years. For the main locations FOM and tongue and for N‐stage, our findings show, that for FOM the effect of the number of pack years is positive and the effect for female gender is negative, but neither effect was statistically significant. But the interaction effect of female gender on pack years is negative with statistical significance (*p* = .037). For patients with tongue cancer, we see the opposite. The effect of pack years is negative and the interaction effect of female gender on pack years is positive, but without reaching statistical significance (Figure 3). We also fitted a logistic model to predict lymph node positivity (N+) with pack years and gender. We see that the effect of pack years is positive and the effect of the female gender is negative for lymph node‐positive status. The interaction effect of the female gender on pack years is positive. All the mentioned effects for the association with lymph node positivity did not reach statistical significance. This was similar in male and female groups (Figure S5 and Table S2).

## 4 DISCUSSION

The purpose of this study was to analyze demographic and clinical data to get information on how gender and risk profile, in relation to smoking and alcohol consumption, influence the clinical presentation of OSCC. We show that men and women in the context of lifestyle demonstrate different patterns of risk. Women comprised more NSND in our study, which replicates results from different studies, which, in turn, demonstrates a higher proportion of women without smoking and alcohol consumption with oral cavity tumors (Farshadpour et al., 2007; Kruse et al., 2010; Moyses et al., 2013). The majority of men present at least one risk factor and represent the majority of SD. Furthermore, we see differences between men and women for age, tumor location, and N‐stage. In the case of T‐and UICC‐stage, there was a tendency seen but without reaching statistical significance.

In our analysis of the lifestyle risk factors we see the same; differences for NSND and SD in age, tumor location and N‐stage and a tendency in T‐stage and UICC‐stage. In view of that, it seems that the differences in men and women are caused by the different risk profiles.

If we look at the same risk profiles within men and women, some similarities were recognizable. Female smokers smoke as intensely as male smokers with the same amount of pack years. Poveda‐Roda et al. (2010) described no difference of gender in the number of cigarettes smoked daily, but they saw for females a shorter duration of smoking than males before the presentation of cancer. This confirmed our results but we did not record the duration of smoking up to the diagnosis of OSCC in our retrospective study. Like our results, Mueller et al. (2008) reported in a 35‐year retrospective study that women were approximately 5 years older at diagnosis than men. This was confirmed by Udeabor et al. (2012). Furthermore, distinct risk groups differ in age. Our results show that SD patients were younger at diagnosis than the other groups. Different studies describe younger patients for smokers compared with non‐smokers (Koo et al., 2013; Moyses et al., 2013; Poveda‐Roda et al., 2010; Schmidt et al., 2004). But additionally, we have demonstrated that within the same risk groups there is no age difference between males and females, that is, although females were older at diagnosis than males, with the same risk profile this effect disappears. It is therefore more likely that females are older at diagnosis because they are mostly represented in the NSND group.

In the present study, SD patients developed more *N* ≥ 2 stage than NSND patients. SD patients presented also with higher UICC‐staging in comparison to the other risk groups. Others have shown that stage IV tumors are more present in smokers than in non‐smokers as well. Poveda‐Roda et al. (2010) and Moyses et al. (2013) saw more node involvement in SD patients, and SD patients presented it at a later pathological stage compared to NSND. Smokers show less well‐differentiated tumors than NSND do. Dahlstrom et al. (2008) reported that the majority of NSND patients with oral cavity carcinoma had well‐differentiated tumors. Others report that males were predominantly affected by moderately and poorly differentiated tumors, whereas females presented mostly with moderately and well‐differentiated tumors (Pires et al., 2013). This partly confirms our results, but the degree of tumor differentiation did not reach significant values in the analysis of gender and of the different risk groups.

We additionally investigate the effect on T‐, N‐, and UICC‐stage and differentiation with the risk factors and gender. For smoking, there are contrary gender effects seen for T‐, N‐, and UICC‐stage. This shows that even if the lifestyle factors are taken into account, clear differences exist between the sexes, and the same effects cannot be seen with the same risk behavior alone. The different risk behavior alone cannot be the cause of the gender differences in tumor characteristics.

Men and women and NSND and SD showed different locations in univariate analysis. Women represented a larger proportion of tongue cancer cases than men did, whereas the main location was still FOM in both sexes. We saw more tongue cancer in NSND patients. There were more cancers of FOM in SD patients. This correlates with the findings of Dahlstrom et al., who showed that NSND oral cavity cancer patients tended to have tongue cancer, but SD patient counterparts had a higher proportion of FOM cancer (Dahlstrom et al., 2008). Our used logistic model confirms the results presented above on the one hand that women develop more tongue cancer and smokers more FOM cancers, and it shows on the other hand that smoking women tend to develop FOM cancers and not tongue cancers. In this way, someone could conclude that if women behave like men they tend to develop cancers at locations like men and that there must be a dose‐effect for smokers in the expression of the location.

So we looked closer at the effect of the pack years for FOM and tongue cancers regarding gender. As expected, the effect of pack years was positive for FOM and negative for tongue cancer. But the interaction effect of female gender on pack years is contrary to what one would have expected. The more pack years women have the less FOM cancer they develop and vice versa in tongue cancer. For men the curves make more sense, the more pack years men have the more FOM cancer they develop. For N‐stage the effects of pack years were as expected for men and women and also confirm the results presented above.

Oral cancer is described as the third‐most significant association between smoking and cancer, following lung cancer and laryngeal cancer (Poveda‐Roda et al., 2010). On the flipside, despite a decline in female smoking prevalence, female incidence rates of lung, laryngeal, and oral cavity cancers increased in most parts of Europe (Lortet‐Tieulent et al., 2015). In the United States, an increasing trend in tongue cancer has been observed in young females, often without risk factors (Chi et al., 2015). The etiology of OSCC in NSND is still unclear. Besides smoking and alcohol consumption, human papilloma virus (HPV), lack of fruits and vegetables, periodontal disease, genetic factors, and premalignant lesions were seen as further risk factors (Petti, 2009; Poveda‐Roda et al., 2010; Shaw & Beasley, 2016). But different studies describe the incidence of HPV in oral tongue carcinoma and OSCC in general as low and unlikely to play a major role in the etiology (Dahlgren et al., 2004; Iyengar et al., 2014; Salem, 2010; Castellsagué et al., 2016; S3 Guideline Diagnosis and Therapy of Oral Squamous Cell Carcinoma Long Version 3.0, 2021).

Environmental tobacco smoking (ETS) is also being discussed as a risk factor for head and neck cancer, with a dose‐dependent increased risk (Zhang et al., 2000). Dahlstrom et al. (2008) also report that ETS may contribute to cancer of the head and neck in NSND women. Furthermore, Koo et al. saw in ETS a possible risk factor with worse disease‐specific mortality and a worse prognosis for elderly female NSND patients and discussed etiological and genetic differences between the NSND and SD groups, resulting in more locally aggressive disease or an increased likelihood of nodal and distant spread (Koo et al., 2013). In our retrospective study, we did not collect data about ETS. To have a better understanding of the etiology of OSCC in NSND patients investigating the influence of ETS is certainly worth considering.

All in all, there still seem to be some unclear facts about the development of OSCC in NSND patients and between men and women. In this way, further research and gender‐specific investigations are necessary.

There are further limitations that have to be pointed out. Firstly, this was a retrospective study. Facts are dependent on accurate documentation. Especially for the registration of the risk factors, there could be a lack of information as far as the risk history is concerned. For example, current non‐smokers could be former smokers. We also did not collect information about the amount of ETS. In the retrospective setting of the study, it is difficult to differentiate from that point of view. Furthermore, in this study, there were no investigations done for viral influences and there are no statements to HPV and p16 status because the influence is small in OSCC and is therefore not the subject of this study. However, an influence cannot be ruled out. Secondly, this study was conducted at a single institution, so external validity is limited. Thirdly, the small number of smoking women, especially the small amount of registered female pack years limits the validity of the analysis.

Prospective studies with larger numbers of patients and a more precise recording of the risk factors are required to eliminate these limitations and investigate this further.

## 5 CONCLUSION

Some but not all differences in the development of OSCC for men and women are explainable by the respective difference in lifestyle behavior. However, the different tumor site distribution is not explainable by lifestyle alone. There must be other reasons for that. For men, there were more consistent results, but for women, there are still some unexplainable facts. Some further investigations are necessary to find explanations for the obvious differences between men and women in developing OSCC. If these could be explained, better and more patient‐specific treatment could be developed considering these differences in gender.

## CONFLICT OF INTERESTS

The authors declare that there are no conflict of interests.

## AUTHOR CONTRIBUTIONS

*Conception and design, acquisition of data, analysis and interpretation of data, drafting the article, revising the article, and final approval*: Susanne Wolfer. *Acquisition of data, revising the article, and final approval*: Annika Kunzler, Tatjana Foos, and Cornelia Ernst. *Statistical analysis and interpretation of data, revising the article, and final approval*: Andreas Leha. *Revising the article and final approval*: Stefan Schultze‐Mosgau.

## Supporting information

Supporting information.

## Data Availability

The data that support the findings of this study are available on request from the corresponding author. The data are not publicly available due to privacy or ethical restrictions.
