# Supplementary material for: Gender and risk‐taking behaviors influence the clinical presentation of oral squamous cell carcinoma
Source: Clin Exp Dent Res. 2022 Jan 5;8(1):141–51. doi: 10.1002/cre2.523 (PMC8874093; doi:10.1002/cre2.523)
Supplement: Supplementary file 1 — Supporting information. [file CRE2-8-141-s001.docx]

Appendix / supplemented material

**Supplemental Figure S1:**

**Different risk-taking behavior according to the T-stage divided for males and females: There is a tendency for less T1 cases in SD patients than in NSND patients, but with no clear differences in gender.** Abbreviations: NSND = non smokers and non drinkers, SND = smokers and non drinker, NSD = non smoker and drinker, SD = smoker and drinker

**Supplemental Figure S2:**

**Different risk-taking behavior according to the N-stage divided for males and females: There are more N>2 cases in SD patients than in NSND patients, but with no differences in gender.** Abbreviations: NSND = non smokers and non drinkers, SND = smokers and non drinker, NSD = non smoker and drinker, SD = smoker and drinker

**Supplemental Figure S3:**

**Different risk-taking behavior according to the UICC-stage divided for males and females: There is a tendency for more UICC-stage IV cases in SD patients than in NSND patients, but with no clear differences in gender.** Abbreviations: NSND = non smokers and non drinkers, SND = smokers and non drinker, NSD = non smoker and drinker, SD = smoker and drinker

**Supplemental Figure S4:**

**Smoker and non smoker in FOM and in tongue cancer divided in gender. (A) There are more FOM cancers in smokers than in non smoker as well in male as in female. (B) For tongue cancer we see the opposite, more tongue cancer in non smoker than in smoker, again without differences in gender.** Marginal model predictions when modelling FOM and tongue by groups. P-values are from pairwise contrast tests. Abbreviations: m = male; f = female

**Supplemental Figure S5:**

**Pack years according to the N-positive-stage divided in gender. The interaction effect of pack years is positive for both male and female. (without statistical significance).** With increasing py there are more N-positive cases in smoking men. For women who smoke you can see the same effect. (logistic regressions model); Abbreviations: m = male; f = female, N+ = positive nodal stage, py = pack years (1 py = regular smoking 20 cigarettes (1 pack)/day for 1 year) ; For statistics see supplement Table S3.

| **location** | **term** | **level** | **term 2** | **level 2** | **effect** | **estimate** | **95% CI** | **p-value** |
| --- | --- | --- | --- | --- | --- | --- | --- | --- |
|  | intercept |  |  |  |  | -0.685 | -1.274; -0.128 | 0.018 |
|  | sex | m |  |  |  |  |  |  |
| **FOM** |  | f |  |  | **-** | -0.603 | -1.505; 0.262 | 0.178 |
|  | drinking | 0 |  |  |  |  |  |  |
|  |  | 1 |  |  | + | 0.425 | - 0.149; 1.001 | 0.146 |
|  | smoking | 0 |  |  |  |  |  |  |
|  |  | 1 |  |  | + | 0.647 | - 0.035; 1.348 | 0.066 |
|  | sex | f | drinking | 0 |  |  |  |  |
|  |  | f |  | 1 | - | - 0.565 | -2.303; 1.111 | 0.511 |
|  |  | f | smoking | 0 |  |  |  |  |
|  |  | f |  | 1 | + | 0.684 | - 0.622; 2.015 | 0.306 |
|  | intercept |  |  |  |  | -0.882 | -1.517; -0.298 | 0.004 |
|  | sex | m |  |  |  |  |  |  |
| **tongue** |  | f |  |  | + | 0.274 | -0.559; 1.119 | 0.520 |
|  | drinking | 0 |  |  |  |  |  |  |
|  |  | 1 |  |  | - | - 0.484 | - 1.188; 0.217 | 0.175 |
|  | smoking | 0 |  |  |  |  |  |  |
|  |  | 1 |  |  | - | - 0.253 | - 1.019; 0.535 | 0.521 |
|  | sex | f | drinking | 0 |  |  |  |  |
|  |  | f |  | 1 | + | 0.583 | -1.609; 2.527 | 0.566 |
|  |  | f | smoking | 0 |  |  |  |  |
|  |  | f |  | 1 | - | - 0.605 | - 2.179; 0.821 | 0.423 |

**Supplemental Table S1: Analysis of risk-taking behavior and tumor main locations FOM and tongue in accordance with gender. See the opposite effects in FOM and tongue.** Model coefficients from a model when modelling FOM and Tongue by gender and risk behavior (logistic regression model); Abbreviations: FOM = Floor of the mouth; m = male; f = female; CI = confidence interval

Within this model

**FOM:** The effect of sex [f] is negative. The effect of smoking [1] is positive. The effect of dinking [1] is positive. The interaction effect of smoking [1] on sex [f] is positive. The interaction effect of drinking [1] on sex [f] is negative.

**Tongue:** The effect of sex [f] is positive. The effect of smoking [1] is negative. The effect of drinking [1] is negative. The interaction effect of smoking [1] on sex [f] is negative. The interaction effect of drinking [1] on sex [f] is positive.

| Parameter | Term 1 | Level 1 | Term 2 | Level 2 | estimate | 95% CI | p-value |
| --- | --- | --- | --- | --- | --- | --- | --- |
| T-stage | drinking | 0 |  |  |  |  |  |
|  |  | 1 |  |  | -0.002 | -0.520; 0.516 | 0.995 |
|  | smoking | 0 |  |  |  |  |  |
|  |  | 1 |  |  | 0.510 | -0.111; 1.122 | 0.110 |
|  | sex | m | drinking | 1 |  |  |  |
|  |  | f |  | 0 |  |  |  |
|  |  | m | drinking | 0 |  |  |  |
|  |  | f |  | 1 | 0.860 | -0.675; 2.429 | 0.272 |
|  |  | m | smoking | 1 |  |  |  |
|  |  | f |  | 0 |  |  |  |
|  |  | m | smoking | 0 |  |  |  |
|  |  | f |  | 1 | -1.154 | -2.377; 0.030 | 0.059 |
| N-stage | drinking | 0 |  |  |  |  |  |
|  |  | 1 |  |  | 0.363 | -0.178; 0.908 | 0.190 |
|  | smoking | 0 |  |  |  |  |  |
|  |  | 1 |  |  | 0.392 | -0.267; 1.072 | 0.249 |
|  | sex | m | drinking | 1 |  |  |  |
|  |  | f |  | 0 |  |  |  |
|  |  | m | drinking | 0 |  |  |  |
|  |  | f |  | 1 | 0.688 | -0.985; 2.347 | 0.409 |
|  |  | m | smoking | 1 |  |  |  |
|  |  | f |  | 0 |  |  |  |
|  |  | m | smoking | 0 |  |  |  |
|  |  | f |  | 1 | -0.671 | -1.999; 0.580 | 0.304 |
| UICC-stage | drinking | 0 |  |  |  |  |  |
|  |  | 1 |  |  | 0.254 | -0.272; 0.780 | 0.342 |
|  | smoking | 0 |  |  |  |  |  |
|  |  | 1 |  |  | 0.425 | -0.194; 1.047 | 0.178 |
|  | sex | m | drinking | 1 |  |  |  |
|  |  | f |  | 0 |  |  |  |
|  |  | m | drinking | 0 |  |  |  |
|  |  | f |  | 1 | 0.244 | -1.276; 1.802 | 0.752 |
|  |  | m | smoking | 1 |  |  |  |
|  |  | f |  | 0 |  |  |  |
|  |  | m | smoking | 0 |  |  |  |
|  |  | f |  | 1 | -0.935 | -2.110; 0.223 | 0.115 |
| differentiation | drinking | 0 |  |  |  |  |  |
|  |  | 1 |  |  | -0.155 | -0.761; 0.444 | 0.613 |
|  | smoking | 0 |  |  |  |  |  |
|  |  | 1 |  |  | -0.438 | -1.176; 0.287 | 0.240 |
|  | sex | m | drinking | 1 |  |  |  |
|  |  | f |  | 0 |  |  |  |
|  |  | m | drinking | 0 |  |  |  |
|  |  | f |  | 1 | -0.221 | -1.968; 1.565 | 0.805 |
|  |  | m | smoking | 1 |  |  |  |
|  |  | f |  | 0 |  |  |  |
|  |  | m | smoking | 0 |  |  |  |
|  |  | f |  | 1 | -0.018 | -1.322; 1.303 | 0.979 |

**Supplemental Table 2: Analysis of T-stage, N-stage, UICC-stage and differentiation in accordance to risk-taking behavior and gender.** Model coefficients from a cumulative link (proportional odds) model when modelling T, N, UICC and differentiation by gender and risk behavior; Abbreviations: m = male; f = female; CI = confidence interval

| **tumor-site** | **term 1** | **level 1** | **term2** | **level 2** | **effect** | **estimate** | **95% CI** | **p-value** |
| --- | --- | --- | --- | --- | --- | --- | --- | --- |
|  | intercept |  |  |  |  | -0.259 | -1.171; 0.626 | 0.570 |
| **FOM** | sex | m |  |  |  |  |  |  |
|  |  | f |  |  | **+** | 3.332 | 0.032; 7.629 | 0.073 |
|  | py |  |  |  |  | 0.016 | -0.008; 0.042 | 0.196 |
|  | py |  | sex | m |  |  |  |  |
|  | py |  |  | f | **-** | -0.115 | -0.245; -0.018 | 0.037 |
|  | intercept |  |  |  |  | -0.425 | -1.514; 0.690 | 0.447 |
| **tongue** | sex | m |  |  |  |  |  |  |
|  |  | f |  |  | **-** | -3.525 | -10.037; 0.627 | 0.163 |
|  | py |  |  |  |  | -0.026 | -0.062; 0.004 | 0.114 |
|  | py |  | sex | m |  |  |  |  |
|  | py |  |  | f | **+** | 0.094 | -0.022; 0.245 | 0.142 |
|  | intercept |  |  |  |  | -0.628 | -1.547; 0.256 | 0.170 |
| **N stage** | sex | m |  |  |  |  |  |  |
|  |  | f |  |  | **´-** | -3.803 | -10.053; 0.224 | 0.123 |
|  | py |  |  |  |  | 0.018 | -0.006; 0.043 | 0.159 |
|  | py |  | sex | m |  |  |  |  |
|  | py |  |  | f | **+** | 0.073 | -0.035; 0.219 | 0.239 |

**Supplemetal Table S3: Analysis of the pack years effect on tumor location FOM and tongue and on N-stage with respect of gender.** **See the opposite effects in FOM and tongue.** Model coefficients from a logistic model when modelling FOM, Tongue and n-stage by pack years in interaction with gender; Abbreviations: FOM = Floor of the mouth; m = male; f = female; CI = confidence interval; py = pack yea
